# P-40. Characterization of ICU-Acquired Bloodstream Infections in a High-Complexity Institution in Bogotá, Colombia since 2017-2024

**DOI:** 10.1093/ofid/ofaf695.269

**Published:** 2026-01-11

**Authors:** Sung Jae Cho, Patricia Reyes Pabon, Jose Antonio Rojas Gambasica

**Affiliations:** Clínica Universitaria Colombia, Bogota, Distrito Capital de Bogota, Colombia; Clínica Universitaria Colombia, Bogota, Distrito Capital de Bogota, Colombia; Clínica Universitaria Colombia, Bogota, Distrito Capital de Bogota, Colombia

## Abstract

**Background:**

Objective To analyze the evolution of the incidence, etiology, and antimicrobial resistance of intensive care unit (ICU)-acquired blood stream infections (BSIs) during the prepandemic, pandemic, and postpandemic periods (2017–2024), evaluating their impact on in-hospital mortality.

**Figure 1:**
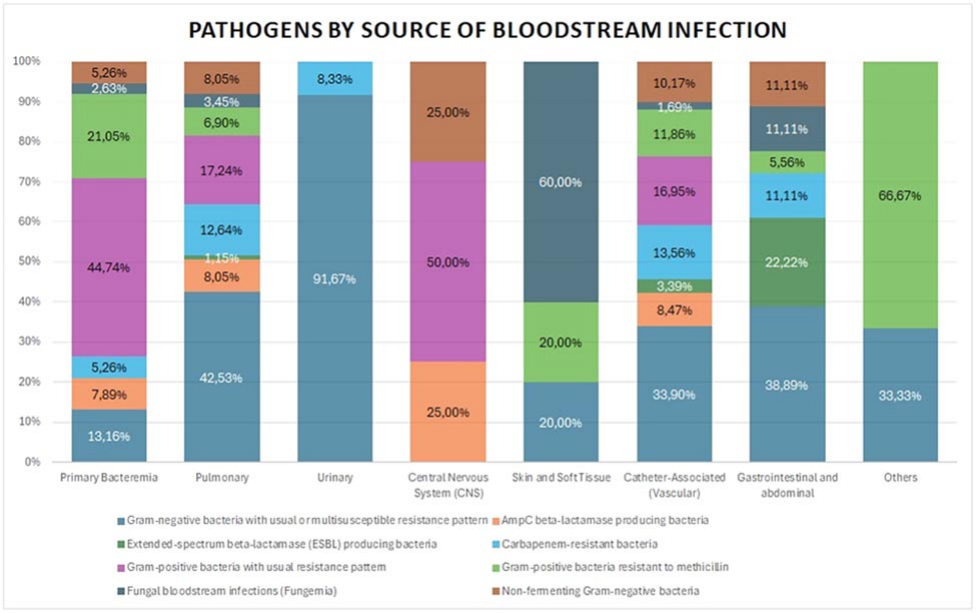
Pathogens by Source of Bloodstream Infection

**Table 1:**
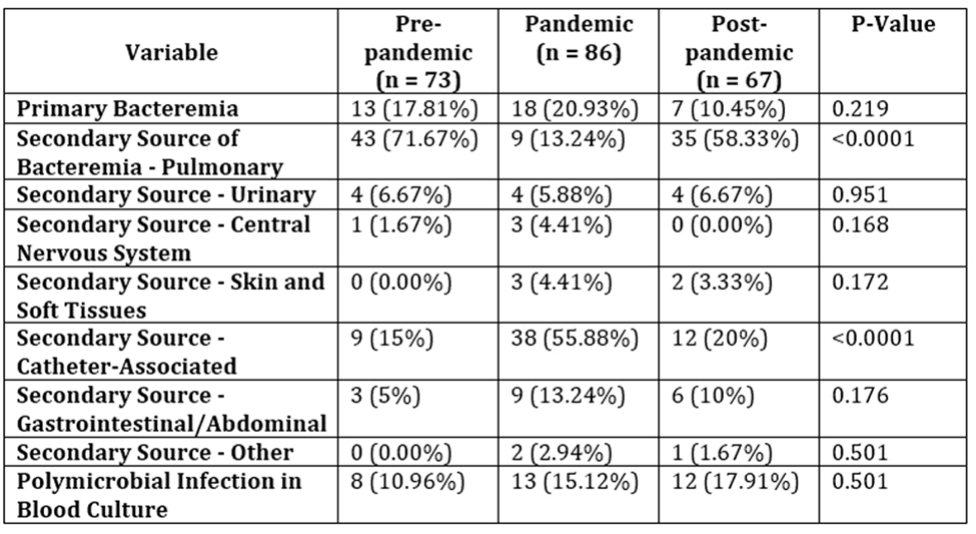
Characteristics of bloodstream infection

**Methods:**

A retrospective observational study was conducted, including 226 patients over 18 years old with (ICU)-acquired BSI from Clínica Universitaria Colombia. Demographic, clinical, microbiological, and mortality variables were analyzed. Univariate and bivariate statistical analyses were used to identify significant differences.

**Table 2:**
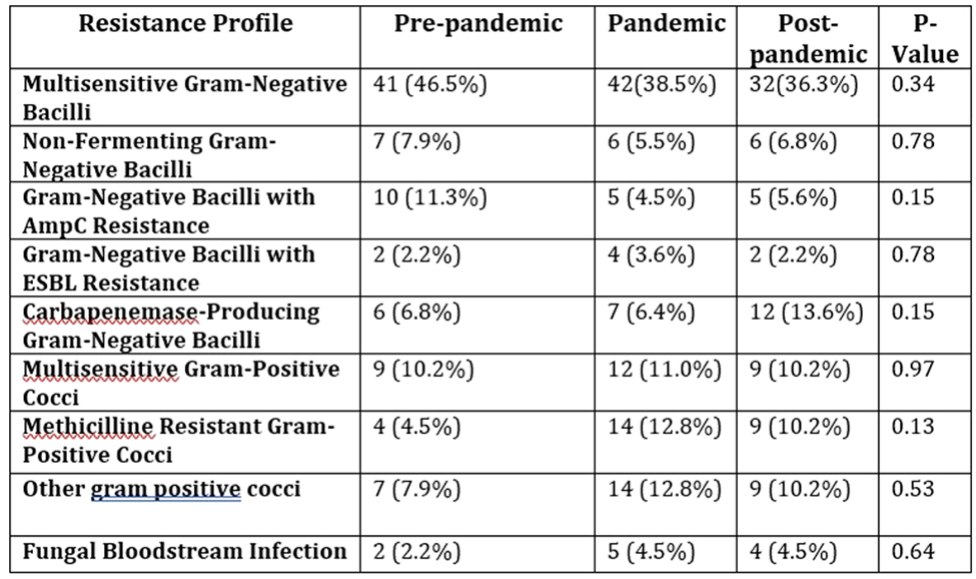
Antimicrobial Resistance profile of identified pathogens

**Results:**

During the SARS-CoV-2 pandemic, the incidence of ICU-acquired infections increased to 1.08%, compared to the prepandemic period 0.72% and postpandemic period 0.64%. Catheter-associated bloodstream infections were the most prevalent during the pandemic period, predominantly caused by methicillin-resistant gram-positive cocci. An increasing trend in the prevalence of carbapenem-resistant organisms was observed in the post-pandemic period (from 6.8% to 13.6%) compared to previous periods. No significant differences were observed in the overall mortality of patients with ICU-acquired BSIs across the periods. An increase in the consumption of antibiotics against methicillin-resistant gram-positive cocci and ceftazidimeavibactam was observed.

**Conclusion:**

The findings demonstrate an increased incidence of ICU-acquired BSIs during the pandemic, along with a rise in carbapenem resistance in the post-pandemic period. These results highlights the need to adjust empirical antimicrobial strategies, reinforce antimicrobial stewardship programs, and enhance infection control measures in critical care setting.

**Disclosures:**

All Authors: No reported disclosures

